# Cell Metabolomics Reveals the Potential Mechanism of Aloe Emodin and Emodin Inhibiting Breast Cancer Metastasis

**DOI:** 10.3390/ijms232213738

**Published:** 2022-11-08

**Authors:** Guorong Cheng, Zhiqiang Liu, Zhong Zheng, Fengrui Song, Xiaoyu Zhuang, Shu Liu

**Affiliations:** 1 State Key Laboratory of Electroanalytical Chemistry & Jilin Province Key Laboratory of Chinese Medicine Chemistry and Mass Spectrometry & National Center of Mass Spectrometry in Changchun, Changchun Institute of Applied Chemistry, Chinese Academy of Sciences, Changchun 130022, China; 2 Experiment Center for Science and Technology, Shanghai University of Traditional Chinese Medicine, Shanghai 201203, China

**Keywords:** aloe emodin (AE), emodin (EMD), cell metabolomics, breast cancer, metastasis

## Abstract

Metastasis is one of the main obstacles for the treatment and prognosis of breast cancer. In this study, the effects and possible mechanisms of aloe emodin (AE) and emodin (EMD) for inhibiting breast cancer metastasis were investigated via cell metabolomics. First, a co-culture model of MCF-7 and HUVEC cells was established and compared with a traditional single culture of MCF-7 cells. The results showed that HUVEC cells could promote the development of cancer cells to a malignant phenotype. Moreover, AE and EMD could inhibit adhesion, invasion, and angiogenesis and induce anoikis of MCF-7 cells in co-culture model. Then, the potential mechanisms behind AE and EMD inhibition of MCF-7 cell metastasis were explored using a metabolomics method based on UPLC-Q-TOF/MS multivariate statistical analysis. Consequently, 27 and 13 biomarkers were identified in AE and EMD groups, respectively, including polyamine metabolism, methionine cycle, TCA cycle, glutathione metabolism, purine metabolism, and aspartate synthesis. The typical metabolites were quantitatively analyzed, and the results showed that the inhibitory effect of AE was significantly better than EMD. All results confirmed that AE and EMD could inhibit metastasis of breast cancer cells through different pathways. Our study provides an overall view of the underlying mechanisms of AE and EMD against breast cancer metastasis.

## 1. Introduction

The metastasis of malignant tumors is the main cause of postoperative recurrence and cancer-related death. Tumor metastasis is a complex multi-step process, which involves uncontrolled growth of early carcinoma in situ, tumor angiogenesis, loss of intercellular adhesion, acquisition of migration and invasion ability, the dissemination of cancer cells from the primary tumor into the extracellular matrix (ECM), entrance into blood vessels, adhesion of cancer cells to the vascular or lymphatic endothelial cells, possibly the transmigration of cancer cells through the endothelium (intravasation and/or extravasation), and finally, the formation of a secondary tumor in a distant targeted organ [1]. Vascular endothelial cells, as a part of the tumor microenvironment, play an important role in the process of tumor invasion and metastasis [2]. It has been reported that adhesion molecules on the surface of endothelial cells can bind to integrin proteins on the surface of cancer cells; thereby, cancer cells can be fixed on the blood vessel wall [3]. The specific growth factors released by endothelial cells in the tumor microenvironment can transform inert cancer cells into more invasive cells, making them more tumorigenic, invasive, and chemoresistant [4]. On the contrary, TNF-α and other factors secreted by cancer cells can stimulate the skeleton rearrangement of endothelial cells, making them shrink and form gaps, so that cancer cells can more easily pass through and reach target organs [5]. Therefore, the co-culture of endothelial cells and cancer cells can better reflect the real growth environment of tumors than cultures of cancer cells alone.

Aloe emodin (AE) and emodin (EMD) are isomers and natural anthraquinone compounds that are found in in traditional Chinese medicine ingredients, such as *Rheum palmatum L*., *Polygonum multiflorum* Thunb, and *Aloe vera*. Studies have shown that AE and EMD can inhibit the invasion and metastasis of various cancers, such as tongue cancer [6], colon cancer [7,8], and breast cancer [9,10]. AE inhibit the migration/angiogenesis of colon cancer cells by reducing the DNA binding activity of NF-κB and then down-regulating the expression of MMP-2, MMP-9, RhoB, and VEGF [8]. AE can also down-regulate the expression of MMP-2 through the p38-dependent MAPK–NF-κB pathway, thereby inhibiting the invasive ability of tumor cells [11]. EMD can inhibit the metastasis of liver cancer by downregulating the expression of CXCR4 [12]. Moreover, EMD can also inhibit epithelial mesenchymal transformation through Wnt/beta-Catenin pathway, thereby inhibiting the invasion and migration of colon cancer cells [7]. However, these studies focus on the effect of AE or EMD on the metastasis of tumor cells cultured alone. It is unclear whether AE or EMD still has inhibitory effect when tumor cells are co-cultured with endothelial cells.

In this work, the co-culture model of breast cancer cells (MCF-7) and human umbilical vein endothelial cells (HUVEC) was established and compared with a traditional single-culture of MCF-7 cells. Then, the effects of AE and EMD on adhesion, invasion, angiogenesis, and anoikis of breast cancer cells were studied by the co-culture model. Furthermore, the mechanism of AE and EMD inhibiting breast cancer metastasis was studied via cell metabolomics.

## 2. Results

### 2.1. Metabolomic Comparison of Single-Culture and Co-Culture MCF-7 Cells

**Figure 1:**
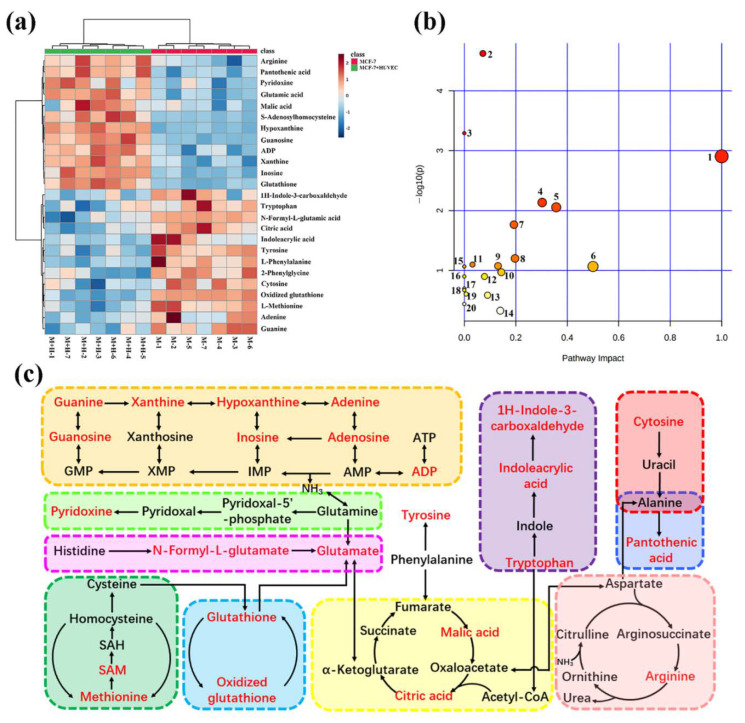
Heat map of single-culture and co-culture MCF-7 cells to visualize the abundance of biomarkers in each group. Each row represents a potential biomarker, each column represents a sample, and the change of color represents the change of biomarker content (**a**). Metabolic pathways affected by the co-culture model of MCF-7 cells. The meaning of numbers is as follows: 1. phenylalanine, tyrosine and tryptophan biosynthesis; 2. purine metabolism; 3. aminoacyl-tRNA biosynthesis; 4. glutathione metabolism; 5. phenylalanine metabolism; 6. D-glutamine and D-glutamate metabolism; 7. arginine biosynthesis; 8. alanine, aspartate and glutamate metabolism; 9. cysteine and methionine metabolism; 10. arginine and proline metabolism; 11. glyoxylate and dicarboxylate metabolism; 12. vitamin B6 metabolism; 13. citrate cycle (TCA cycle); 14. tyrosine metabolism; 15. nitrogen metabolism; 16. ubiquinone and other terpenoid–quinone biosynthesis; 17. butanoate metabolism; 18. histidine metabolism; 19. pantothenate and CoA biosynthesis; 20. porphyrin and chlorophyll metabolism (**b**). Correlation networks of potential biomarkers. The red-marked metabolites were regarded as the potential metabolites (**c**).

To dissect the effect of HUVEC cells on MCF-7 cells in the co-culture system, metabolomic analysis was performed. First, the effect of the co-culture model on the changes of polar metabolites in MCF-7 cells was studied by untargeted cell metabolomics. Figure S1 shows the extracted ion chromatograms of polar metabolites of single-culture and co-culture MCF-7 cells in positive and negative ion modes. Also, 25 biomarkers were identified on the basis of UPLC-Q-TOF/MS multivariate statistical analysis (Table S1, Figures S2 and S3), most of which were small molecular acids and purine metabolites. Figure 1a showed the abundance change of each potential biomarker. There was obvious separation between the MCF-7 cell single-culture group and the co-culture groups, and the samples of the same group could be well gathered together. Next, pathway analysis of biomarkers was carried out to reveal the biological impact of these metabonomic data based on MetaboAnalyst (Figure 1b). The results showed that the metabolic pathways of MCF-7 cells affected by co-culture model mainly include purine metabolism, aminoacyl-tRNA biosynthesis, TCA cycle, glutathione metabolism, arginine biosynthesis, glutamine and glutamate metabolism, cysteine and methionine metabolism, vitamin B6 metabolism, etc. Meanwhile, in order to reveal the complex biochemical relationship between these potential biomarkers, the network visualization analysis of metabolic pathways was carried out through Cytoscape (Figure S4).

The untargeted cell metabolomic approach was global metabolite analysis, which lacked specificity. Thus, we used targeted metabolomics to quantitatively analyze metabolites of key metabolic pathways, which further validated the results of untargeted metabolomics. MS parameters and regression equations of each metabolite are shown in Tables S2 and S3. R^2^ of each metabolite was more than 0.99, which showed that the regression equations have good linearity and accuracy of quantitative results. Figure 1c shows typical metabolic pathways, in which the red-marked metabolites were seen as potential biomarkers identified by untargeted metabolomics. Glutathione, methionine cycle, TCA cycle, tryptophan, and purine metabolism were closely related to tumor progression [13,14,15,16,17].

**Figure 2:**
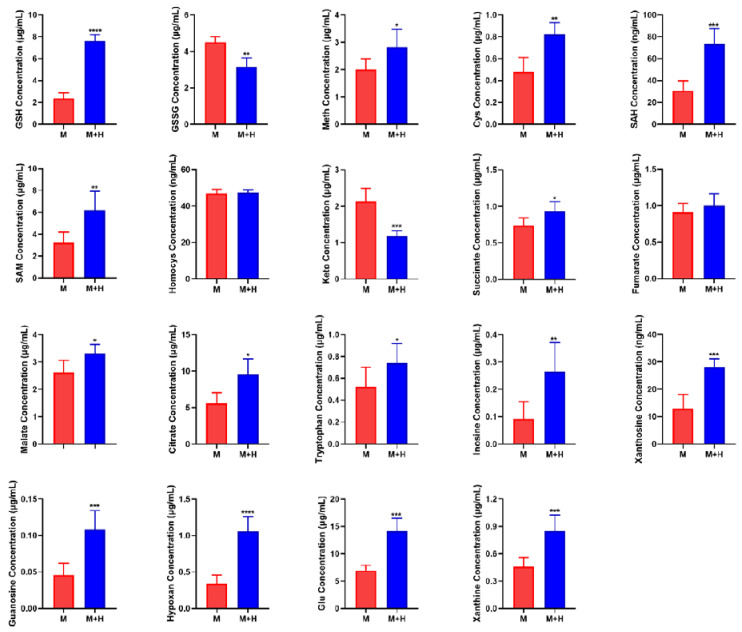
Quantitative analysis of biomarkers. The data are expressed as mean ± SD (*n* = 6). * *p* < 0.05, ** *p* < 0.01, *** *p* < 0.001, **** *p* < 0.0001. Abbreviations are as follows: M represents MCF-7 cell single-culture group, M + H represents the co-culture group of MCF-7 and HUVEC cells. GSH, glutathione; GSSG, oxidized glutathione; Meth, methionine; SAM, S-adenosine methionine; SAH, S-adenosine homocysteine; Cys, cysteine; Hypoxan, hypoxanthine; Homocys, homocysteine; Glu, glutamate; Keto, α-ketoglutarate.

Quantitative analysis of biomarkers was shown in Figure 2. Abnormal glutathione metabolism is closely related to tumor progression. The quantitative results showed that the content of glutathione increased, and the content of oxidized glutathione decreased in the co-culture group, which indicated that HUVEC cells can promote the development of MCF-7 cells to a malignant phenotype. Some studies have shown that methionine cycle activation promotes the occurrence and development of tumors. The quantitative results showed that the contents of cysteine, SAH, SAM, and methionine increased significantly, but not homocysteine, in the co-culture model. The constant content of homocysteine might be attributed to its production rate, which was consistent with its conversion rate to methionine and cysteine. It has been reported that homocysteine is a thiol compound able to inhibit tumor cell proliferation and invasion [18].

The TCA cycle is a metabolic pathway necessary for the production of ATP and precursors used in many biosynthetic pathways. Cancer cells use metabolic rearrangement to maintain their high proliferation rate and energy demand. Therefore, imbalances in TCA cycle flux are often observed in cancer. The identification of enzyme mutations in the TCA cycle of human tumors has shown that this metabolic pathway is directly related to the occurrence of cancer [19]. The quantitative results showed that the content of metabolites in TCA cycle increased, except α-ketoglutarate, in the co-culture model. Recent research reports that the accumulation of α-ketoglutarate can help resist the malignant progression of tumors, and the increase of succinic acid can weaken this resistance [15]. Therefore, we speculate that the increase of α-ketoglutarate and the decrease of succinate can promote the malignant development of MCF-7 cells in the co-culture model. The increase of other metabolites suggests that the TCA cycle becomes more active, which is more conducive to the rapid proliferation of tumors.

Tryptophan metabolism is related to immune response in the tumor microenvironment. Tryptophan metabolites can induce immunosuppression and involve effector immune cell apoptosis. Tryptophan is the key to immune escape [16]. Therefore, the increase of tryptophan is beneficial to tumor progression. Moreover, the quantitative analysis of hypoxanthine, inosine, xanthine, xanthosine, and guanosine in purine metabolic pathway has shown that these purine metabolites were significantly increased in co-culture group compared with the MCF-7 cell single-culture group. It has been reported that high-speed de novo synthesis of purine and pyrimidine can promote tumor proliferation and improve its tumorigenic ability, which is considered to be related to tumor drug resistance and recurrence. Inhibition of purine synthesis may be a promising strategy to overcome gene-heterogeneous disease and treat drug resistance [17].

These results indicated that endothelial cells can promote the development of cancer cells to a malignant phenotype [20,21,22]. The co-culture model of MCF-7 cells and HUVEC cells was closer to the real tumor environment than that of the MCF-7 cell single-culture alone. Therefore, the subsequent experiments were carried out by cell co-culture model.

### 2.2. Cytotoxicity of AE and EMD

**Figure 3:**
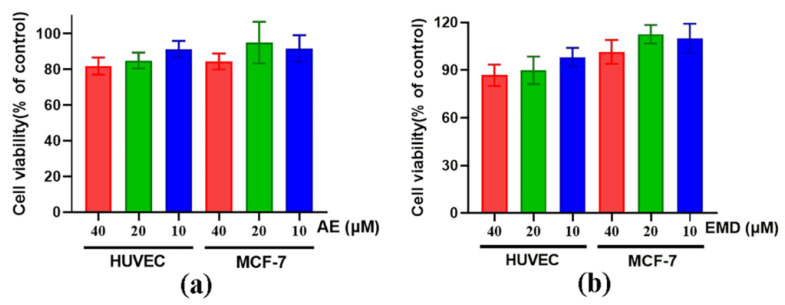
The cytotoxicity of AE (**a**) and EMD (**b**) at different concentrations (40, 20, 10 μM) on HUVEC and MCF-7 cells.

CCK-8 assay was used to evaluate the toxicity of AE and EMD on MCF-7 and HUVEC cells. High, medium, and low concentrations (40, 20, 10 μM) of AE and EMD were selected for cytotoxicity. The results are shown in Figure 3. At the same concentration, AE was slightly more toxic than EMD to MCF-7 and HUVEC cells. Moreover, the survival rates of the two cells treated with different concentrations of AE and EMD were more than 80%. Therefore, these three concentrations were selected for subsequent experiments.

### 2.3. AE and EMD Reduce the Adhesion of MCF-7 Cells to HUVEC Cells

**Figure 4:**
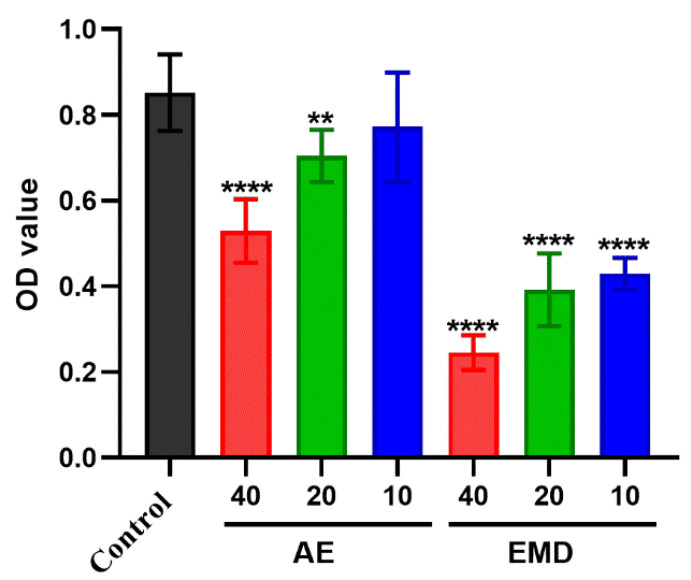
The effects of AE and EMD at different concentrations (40, 20, 10 μM) on the adhesion. The data are expressed as the mean ± SD (*n* = 3). ** *p* < 0.01, **** *p* < 0.0001 compared with control group.

The effects of AE and EMD on the adhesion between MCF-7 cells and HUVEC cells were observed by Rose Bengal staining. It was clear that both AE and EMD could inhibit the adhesion of MCF-7 cells to HUVEC cells (Figure 4). Along with the increase of drug concentration, the absorbance of tumor cells decreased, indicating that the inhibitory effect of the drug became strong. This phenomenon revealed that AE and EMD inhibited the adhesion of MCF-7 cells to HUVEC cells in a dose-dependent manner. Moreover, EMD had a stronger inhibitory effect on adhesion than AE at the same concentration.

### 2.4. AE and EMD Inhibit the Invasion and Metastasis of MCF-7 Cells

**Figure 5:**
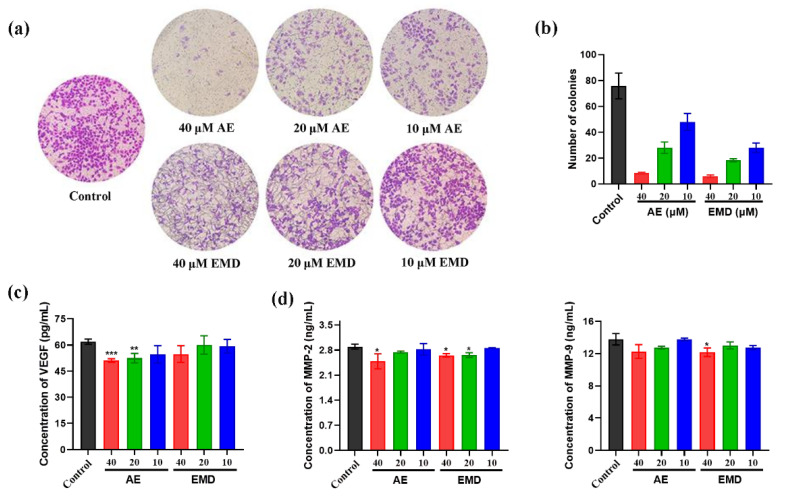
The effects of AE and EMD at different concentrations (40, 20, 10 μM) on the invasion (**a**), soft agar clone (**b**), VEGF secretion (**c**), MMP-2 and MMP-9 secretion (**d**) of MCF-7 cells. The data are expressed as the mean ± SD (*n* = 3). * *p* < 0.05, ** *p* < 0.01, *** *p* < 0.001, compared with control group.

The effects of AE and EMD on invasion of breast cancer were investigated by transwell cell co-culture model. Figure 5a shows that the inhibitory effect of AE on the invasion of MCF-7 cells is significantly better than that of EMD, and the inhibitory effect of AE became better with the increase of concentration. In addition, we simulated the proliferation ability of tumor cells in semi solid state by soft agar cloning assay and studied the effects of AE and EMD on anoikis of MCF-7 cells. Figure 5b shows that AE and EMD could promote the anoikis of MCF-7 cells in a concentration-dependent manner. The promoting effect of EMD was better than that of AE at the same concentration.

Vascular endothelial growth factor (VEGF) is an important factor in regulating angiogenesis. Inhibiting the production of VEGF to target tumor angiogenesis contributes to control tumor growth and metastasis. The results showed that AE could significantly inhibit the secretion of VEGF, while EMD had no significant inhibitory effect (Figure 5c). Moreover, we also measured the effects of AE and EMD on MMP-2 and MMP-9. Figure 5d showed that 40 μM AE could significantly inhibit the secretion of MMP-2. EMD had a good inhibitory effect on MMP-2 and MMP-9, while the inhibitory effect on MMP-2 was weaker than that of AE.

### 2.5. Effects of AE and EMD on Metabolism of MCF-7 Cells

**Figure 6:**
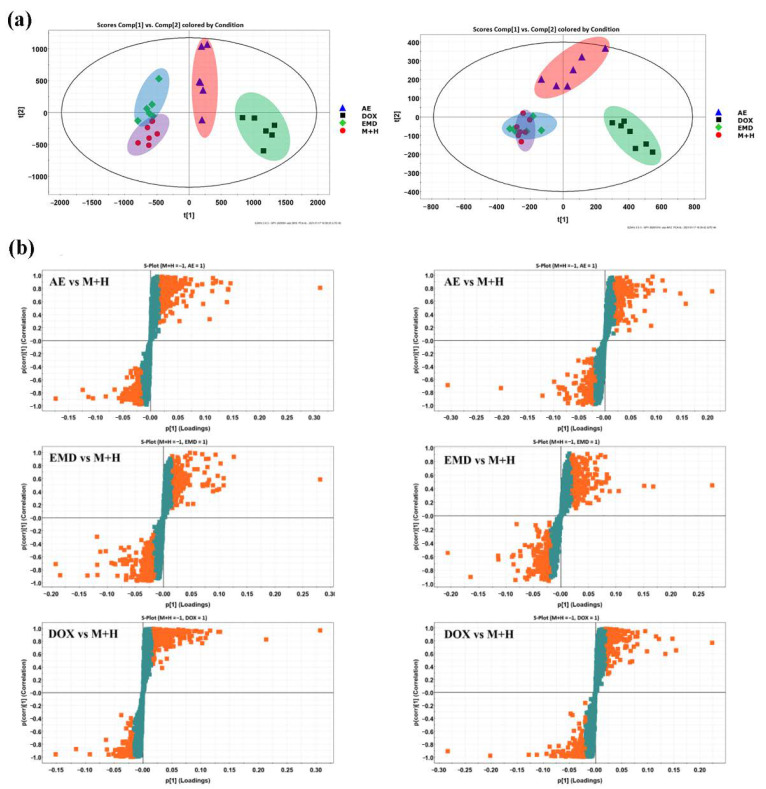
PCA score plots of aloe emodin (AE), emodin (EMD), doxorubicin (DOX), and control group (M + H) in positive (**left**) and negative (**right**) ion mode (**a**). S-polts of metabolic profiling between aloe emodin (AE)/emodin (EMD)/doxorubicin (DOX) and control group (M + H) in positive (**left**) and negative (**right**) ion modes. The orange and green cubes represent metabolites of VIP>1 and VIP<1, respectively (**b**).

The mechanism of AE and EMD inhibiting metastasis of MCF-7 cells was investigated by untargeted cell metabolomic analysis. Doxorubicin (DOX), a common chemotherapeutic drug, was used as the positive control. PCA score plots (Figure 6a) showed that the DOX and AE groups could be separated from the control group in both positive and negative ion mode, while the EMD group could only be separated from the control group in positive ion mode. The results indicated that the intracellular metabolites changed significantly when MCF-7 cells were treated with DOX or AE.

Next, PLS-DA analysis (Figure S5) and S-polts (Figure 6b) were performed to screen the potential biomarkers. The EMD group and control group could be well separated through supervised PLS-DA analysis, although their separation effect was not satisfactory in PCA score plots, especially in negative ion mode. This result indicated that the difference between groups was much greater than that within groups; the metabolism of MCF-7 cells treated with EMD was also disturbed. Good fitting and high prediction capabilities were observed in PLS-DA plots via the high statistical values of R^2^Y and Q^2^, respectively. The parameters were R^2^Y = 0.9989 and Q^2^ = 0.9823 between AE and control groups in the positive ion mode, R^2^Y = 0.9999 and Q^2^ = 0.9451 in the negative ion mode, R^2^Y = 0.9909 and Q^2^ = 0.9025 between EMD and control groups in the positive ion mode, and R^2^Y = 0.9986 and Q^2^ = 0.9186 in the negative ion mode. The metabolites highlighted in orange in S-polts showed high contributions, that is, VIP > 1. After preliminary screening, the metabolites with VIP > 1 and *p* < 0.05 were considered as candidate biomarkers. Subsequently, the biomarkers were further identified by MS/MS secondary fragmentation and comparison with the retention time and accurate molecular weight of standards. Finally, 27 biomarkers were identified in AE group, and these biomarkers were distributed in various metabolic pathways, including polyamine, methionine cycle, glutathione, purine, sphingolipid, TCA cycle, and tryptophan metabolic pathways; 13 biomarkers were identified in the EMD group, involving glutathione metabolism, purine metabolism, methionine cycle, phenylalanine metabolism, sphingolipid metabolism, and TCA cycle (Table 1).

### 2.6. Quantitative Analysis of Biomarkers

**Figure 7:**
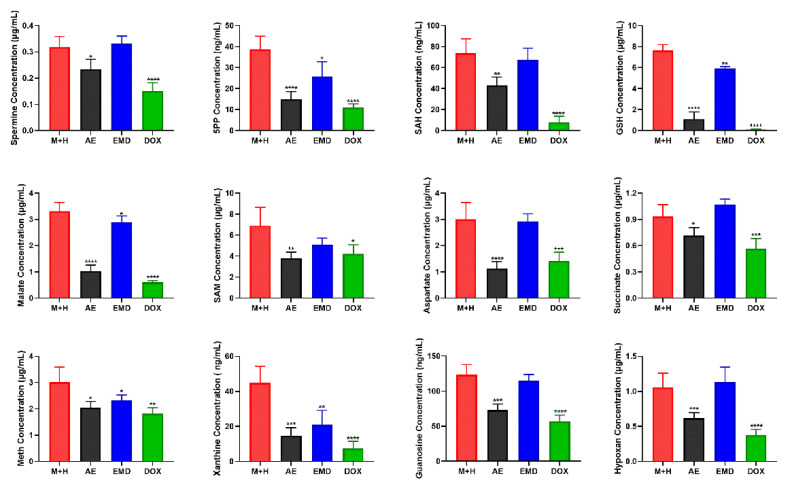
Quantitative analysis of biomarkers. The data are expressed as the mean ± SD (*n* = 6). * *p* < 0.05, ** *p* < 0.01, *** *p* < 0.001, **** *p* < 0.0001 compared with control group.

Untargeted metabolomics consists of global metabolite analysis, which lacks specificity, and the quantitative results may not be accurate enough. Thus, biomarkers of key metabolic pathways were quantitatively analyzed; the results are shown in Figure 7.

Polyamine metabolism is involved in various biological activities of cancer cell progression, including proliferation, differentiation, migration, and apoptosis. Studies have reported that elevated polyamine levels are conducive to tumor metastasis. Thus, reducing the level of polyamine may help to prevent and treat tumor metastasis [23]. Our results showed that AE could significantly reduce the level of spermine, while EMD could not.

Methionine cycle is highly activated in tumor-initiating cells. When this pathway is inhibited, tumor-initiating cells lose their tumorigenicity, indicating that the activation of methionine cycle is closely related to tumorigenesis [24]. The quantitative analysis of SAH, SAM, and methionine in methionine cycle showed that AE and EMD had a down-regulated trend on these three metabolites, but EMD could only significantly reduce the content of methionine.

Glutathione metabolism is very important for maintaining intracellular redox homeostasis. Glutathione can be used as an antidote to combine with antineoplastic, making it lose antitumor effect, producing drug resistance. In addition, abnormal glutathione metabolism can also promote tumor progression. Many studies have reported that increasing the level of glutathione can promote tumor metastasis [13]. As shown in Figure 7, AE can reduce the content of glutathione, which may help to inhibit cancer metastasis.

Purine metabolites are one of the most abundant metabolites in mammals. Purine metabolites can not only be used as building blocks of DNA and RNA, but also provide necessary energy and cofactors to promote cell survival and proliferation. The rapid proliferation of tumor cells requires the energy supply and the rapid synthesis and replication of DNA. Therefore, purine metabolism is very important in the process of carcinogenesis and development [21]. Moreover, the TCA cycle is an essential metabolic pathway for the production of ATP and precursors used in many biosynthetic pathways. It has been reported that the imbalance of the TCA cycle is also closely related to tumorigenesis [19]. Our results showed that AE had a good inhibitory effect on purine metabolic pathways and could significantly downregulate the levels of xanthine, guanine nucleoside, and hypoxanthine. Meanwhile, AE could also significantly reduce the contents of malate and succinate. While EMD demonstrated this activity as well, the effect was poor.

Aspartate is a key nutrient for tumor growth, especially under hypoxia. Tumor proliferation becomes faster when the level of aspartate in vivo is increased, indicating that inhibiting the production or acquisition of aspartate may be an effective anti-tumor strategy [25]. Our results showed that AE could significantly reduce the content of aspartate in MCF-7 cells.

In sum, AE had a good inhibitory effect on polyamine metabolism, methionine cycle, glutathione metabolism, purine metabolism, TCA cycle, and aspartate synthesis. These metabolic pathways provided a new understanding for the mechanism of AE inhibiting breast cancer metastasis. EMD had less effect on metabolites in breast cancer.

## 3. Discussion

Tumor metastasis is a complex multi-step and multi-factor process. The traditional tumor metastasis model is based on a separate cancer-cell culture. However, this culture method is far from the real tumor growth environment, causing the unsatisfactory results in vivo of some effective drugs in vitro. In the present study, the co-culture model of breast cancer cells (MCF-7) and HUVEC cells was established and compared with a traditional single culture of MCF-7 cells via cell metabolomics. Our results showed that HUVEC cells could further enhance glutathione, methionine cycle, TCA cycle, tryptophan, and purine metabolism of MCF-7 cells, which were closely related to tumor progression. This phenomenon supported endothelial cells can promote the development of cancer cells to malignant phenotype. This co-culture model is closer to the real tumor growth environment and is more suitable for studying the metastasis of cancer cells in vitro than traditional single-culture methods.

AE and EMD, two anthraquinone isomers present in various traditional Chinese medicine ingredients, such as *Rheum palmatum L.* and *Polygonum multiflorum* Thunb, have been extensively studied for their anti-tumor metastasis activities. However, few studies have been conducted on the inhibitory effect and mechanism of AE and EMD for tumor metastasis based on the co-culture model. Therefore, in this work, we used the co-culture model to evaluate the effects of AE and EMD on important steps and key factors in breast cancer metastasis, such as adhesion, invasion, angiogenesis, etc. Furthermore, the potential inhibitory mechanism of AE and EMD on MCF-7 cells metastasis was studied based on the co-culture model via cell metabolomics.

When tumor cells shed from the primary site flow with the blood circulation and reach the targeted organ, they need to rely on the adhesion ability of endothelial cells to locate in said organ and then proliferate to form metastasis [26]. Therefore, the adhesion of tumor cells to vascular endothelial cells is very important in the process of tumor invasion and metastasis. Inhibiting the heterogeneous adhesion between them can effectively prevent tumor invasion and metastasis. Our results verified that AE and EMD inhibited the adhesion of MCF-7 cells to HUVEC cells in a dose-dependent manner (Figure 4). In addition, AE and EMD could also inhibit the invasion of and promote anoikis in MCF-7 cells in the co-culture model (Figure 5a,b).

Angiogenesis is essential in the process of tumor growth and metastasis. Blood vessels can not only provide rich nutrition and oxygen for cancer cells, but also discharge toxic waste. In addition, cancer cells can enter the circulatory system, migrate, and establish distant metastatic colonies via vascular transport. Tumor cells can release angiogenic regulatory factors and recruit angiogenic stimulating factors from the extracellular matrix to induce the proliferation, migration, and arrangement of endothelial cells [27]. VEGF is an important factor in regulating angiogenesis. Matrix metalloproteinases (MMPs) are zinc-dependent proteolytic enzymes. MMPs are considered to be the most important effectors in the process of tumor metastasis and angiogenesis, because they can effectively degrade a variety of substrates around tumor cells and vascular basement membrane of endothelial cells [28]. MMP-2 and MMP-9 are important members of MMPs family and are highly expressed in many tumors. They can destroy the histological barrier in the process of tumor cell invasion and play a key role in tumor metastasis [29]. Our results showed that AE could inhibit the secretion of VEGF and MMP-2; in comparison, EMD did not significantly inhibit the secretion of VEGF—only MMP-2 and 9 (Figure 5c,d). The results might be related to the different steps involved in AE and EMD inhibiting MCF-7 cells metastasis.

The potential mechanism of AE and EMD inhibiting breast cancer metastasis was further studied through a combination of untargeted and targeted metabolomics. A total of 27 and 13 biomarkers were detected after AE and EMD intervention, respectively. The results indicated that AE inhibition of breast cancer metastasis could mainly be attributed to polyamine metabolism, methionine cycle, TCA cycle, glutathione metabolism, purine metabolism, and aspartate synthesis. On the contrary, EMD slightly inhibited these metabolic pathways, but the effect was weak.

## 4. Materials and Methods

### 4.1. Materials

AE and EMD were purchased from Chengdu Manster Biotech Co., Ltd. (Chengdu, Sichuan province, China). Citrate, malate, fumarate, succinate, and α-ketoglutarate were purchased from Shanghai Winherb Medical Technology Co., Ltd. (Shanghai, China). S-adenosyl-methionine (SAM), S-adenosyl-L-homocysteine (SAH), homocysteine, xanthine, hypoxanthine, xanthosine, inosine, and guanosine were purchased from Aladdin Chemistry Co. (Shanghai, China). Aspartate (Asp), glutamate (Glu), glutamine (Gln), methionine, cysteine, glutathione (GSH), oxidized glutathione (GSSG), tryptophan, and agarose were purchased from Sigma-Aldrich (St. Louis, MO, USA). CCK-8, treptomycin, penicillin, and trypsin were obtained from Beijing Dingguo Co., Ltd. (Beijing, China). DMEM high-glucose medium and fetal bovine serum were purchased from Gibco (Grand Island, NE, USA) and BI (Kibbutz Beit Haemek, Israel), respectively. Human matrix metalloproteinase 2 (MMP-2), human matrix metalloproteinase 9 (MMP-9), and human vascular endothelial growth factor (VEGF) ELISA kits were purchased from Jianglai Biotechnology Co., Ltd. (Shanghai, China). Matrigel matrix was purchased from BD Biosciences (Franklin Lakes, NJ, USA). HPLC-grade acetonitrile, methanol, chloroform, and formic acid were supplied by Merck (Darmstadt, Germany). Ultrapure water was prepared by a Milli-Q water purification system (Milford, MA, USA).

### 4.2. Cell Culture

Human umbilical vein endothelial cell HUVEC and human breast cancer cells MCF-7 were cultured in DMEM high-glucose medium with 10% fetal bovine serum, 100 μg/mL treptomycin, and 100 U/mL penicillin. Cells were incubated in a humidified atmosphere of 5% CO^2^ at 37 °C.

### 4.3. Cytotoxicity Assay

To determine whether AE and EMD have cytotoxic effect, CCK-8 assay was conducted. HUVEC and MCF-7 cells were seeded into the upper chamber and lower chamber of 24-well transwell plate at the density of 3 × 10^3^/well/200 μL and 6 × 10^3^/well/600 μL, respectively. After cell adhesion, the cells were treated with AE and EMD (40, 20, and 10 μM) for 48 h and the control group was supplemented with the same volume of culture medium. Then the upper chamber was taken out and placed in a new 24-well plate. CCK-8 reagents were added into the upper chamber and lower chamber, respectively, for 2 h. Finally, 100 μL liquid were drawn from each chamber and placed in a 96-well plate, and the absorbance value at 450 nm was measured by TECAN GENios microplate reader (Mannedorf, Switzerland). The changes of absorbance values of upper and lower chambers indicated the inhibitory effect of drugs on the proliferation of HUVEC and MCF-7 cells in separate co-culture systems.

### 4.4. Adhesion Assay

HUVEC cells with 9 × 10^4^/mL were seeded into 96-well plates and cultured for 72 h to make them grow to 100% confluence. Then, 200 μL of 4 × 10^5^/mL MCF-7 cells suspension treated with AE and EMD (40, 20, 10, and 0 μM) for 48 h was added to a 96-well plate full of HUVEC cells, and 6 parallel wells were set in every group. After co-culture for 2 h, the supernatant was sucked away, and MCF-7 cells not adhered to HUVEC cells were washed with fresh culture medium. Then, 100 μL of 0.25% Rose Bengal solution was added and stained for 5 min at 37 °C. Next, the dye solution was sucked and washed with culture medium. After washing, 200 μL of 50% ethanol was added to fixed cells at room temperature for 1 h, and the absorbance value at 570 nm was measured by TECAN GENios microplate reader (Mannedorf, Switzerland). The adhesion ability of MCF-7 cells to HUVEC cells was evaluated by the change of absorbance value of MCF-7 cells.

### 4.5. Invasion Assay

The invasion assay of MCF-7 cells was carried out using a 24-well polycarbonate transwell plate whose upper chambers was covered with matrigel matrix. Then, 200 μL of 2 × 10^4^/well MCF-7 cells suspension containing AE and EMD (40, 20, 10, and 0 μM) without serum was added to the upper chambers of transwell plates. Meanwhile, 600 μL of 6 × 10^4^/well HUVEC cells suspension containing drugs and 10% serum was added to the lower chamber. Then, 48 h later, MCF-7 cells that did not pass through the membrane were gently wiped off with a cotton swab and cells on the back of upper chamber were fixed with 4% paraformaldehyde, followed by 0.1% crystal violet staining. Finally, the effects of different concentrations of AE and EMD on the invasion of MCF-7 cells were observed by inverted microscope.

### 4.6. Activity Determination of MMP-2, MMP-9, and VEGF

MCF-7 and HUVEC cells were seeded into the upper and lower chambers, respectively, of a 24-well transwell plate. After 24 h adhesion, the cells were treated with AE and EMD (40, 20, and 10 μM) for 48 h, and the control group was supplemented with the same volume of culture medium. Then, the supernatants were taken out and placed in a 1.5 mL centrifuge tube. The activities of MMP-2, MMP-9, and VEGF in the supernatant were measured according to the instructions of the corresponding kits.

### 4.7. Soft Agar Colony Formation

Here, 1.5 mL of a lower layer consisting of 0.6% agar in complete DMEM medium with 10% FBS was placed in 6-well plate and permitted to solidify. Then, 5 × 10^3^/well MCF-7 cells treated with AE and EMD (40, 20, 10, and 0 μM) for 48 h were then suspended in a 1.5 mL layer of 0.35% agar in complete DMEM medium and layered on top of the bottom layer. Colonies were observed and counted with an inverted microscope after approximately 2–3 weeks.

### 4.8. Cell Sample Preparation for Metabolomic Analyses

HUVEC and MCF-7 cells were seeded into the upper chamber and lower chamber of a 12-well transwell plate at a density of 2 × 10^4^/mL/0.5 mL and 4 × 10^4^/mL/1.5 mL, respectively. The experiment was divided into MCF-7 cell single-culture group, MCF-7 and HUVEC cell co-culture group, and drug treatment group of co-culture system. After 24 h, the cells were treated with AE and EMD (20 μM) for 48 h, and then the cell metabolomics samples were prepared according to the previous method [30]. MCF-7 cells were resuspended in 0.15 mL ultrapure water after digestion, centrifugation, and collection. Cell samples were lysed by repeated freezing and thawing at −80 °C and 37 °C 3 times. Then, cell lysate was successively added with 0.6 mL ice methanol and 0.45 mL chloroform. After vortex mixing for 30 min, the sample was added with 0.15 mL ultrapure water and stood at −20 °C for 6–8 h, followed by centrifuging at 12,000 rpm for 20 min at 4 °C. The upper layer of the sample was water-soluble substances in MCF-7 cells, and the lower layer was lipids. The upper solution was carefully sucked out, dried under a stream of nitrogen, and stored at −20 °C. The dried samples were re-dissolved with the initial mobile phase for metabolomic analysis.

### 4.9. UPLC-MS Conditions

Untargeted metabolomic analysis was carried out by using a Waters ACQUITY UPLC system (Waters Corp, Milford, MA, USA) interfaced with Q-TOF SYNAPT G2S high-resolution mass spectrometer. Electrospray ion source (ESI) (Waters, Manchester, UK) in the mass spectrometer was conducted in both positive and negative mode in full scan with a mass range of 50–1000 *m*/*z*. Chromatographic conditions: the positive and negative spectrum separation conditions of polar metabolites were the same. An ACQUITYUPLC^®^HSS T3 column (2.1 mm × 50 mm, 1.8 μm) was used for chromatographic separation at 37 °C, and the flow rate was set at 0.3 mL/min. The sample injection volume was 10 μL. The mobile phase was acetonitrile (A) and water containing 0.1% formic acid (B). The gradient elution of the mobile phase was set as follows: 0–3 min, 0% A; 3–5 min, 0–20% A; 5–10 min, 20–50% A; 10–12 min, 50–100% A; 12–15 min, 100% A; 15–17 min, 100–0% A; 17–20 min, 0% A. MS conditions in negative mode were as follows: capillary voltage, 2.0 kV; sampling cone voltage, 30 V; extraction cone voltage, 5.0 V; source temperature, 100 °C; desolvation temperature, 300 °C; desolvation gas, 350 L/h; cone gas, 30 L/h. MS conditions in positive mode were the same as those in negative mode, except that the capillary voltage was 2.8 kV.

Targeted metabolomic analysis was performed by a Shimadzu UPLC–MS system, which consisted of an ultra-high-performance liquid chromatography system (LC-30A) coupled with a triple quadrupole mass spectrometer (LCMS-8050) equipped with an ESI source (Kyoto, Japan). The selection of chromatographic column and mobile phase were the same as above. The elution program in positive mode was set as follows: 0–3 min, 0% A; 3–12 min, 0–50% A; 12–17 min, 50–100% A; 17–20 min, 100% A; 20–21 min, 100–0% A; 21–25 min, 0% A. The elution program in negative mode was set as follows: 0–2 min, 0% A; 2–8 min, 0–50% A; 8–14 min, 50–100% A; 14–17 min, 100% A; 17–18 min, 100–0% A; 18–20 min, 0% A. MS conditions were the same in positive and negative mode, and the optimized operation parameters were as follows: nebulizing gas, 3.0 L/min; drying gas, 10.0 L/min; heating gas, 10.0 L/min; interface temperature, 300 °C; DL temperature, 250 °C; heat block temperature, 400 °C. The precursor ion, product ion, Q1 pre-deviation, collision energy (CE), and Q3 pre-deviation of related compounds were optimized by multiple reactions monitoring (MRM) mode and are listed in Table S1.

### 4.10. Data Processing and Analysis

The MS^E^ data obtained by UPLC-Q-TOF/MS were processed with Progenesis QI software (Waters Corp, Milford, MA, USA), and all data were peak extracted, peak corrected, and normalized. EZinfo 2.0 software was used for principal component analysis (PCA) and orthogonal partial least squares discrimination analysis (OPLS-DA). According to VIP > 1 and *p* < 0.05, the compounds with significant differences among groups were screened and then imported into Progenesis QI software for preliminary identification. These compounds were further identified by comparing and analyzing the retention time and secondary fragments, using HMDB (http://www.hmdb.ca/, accessed on 20 September 2020) and METLIN (http://metlin.scripps.edu/, accessed on 25 September 2020), which were two free biochemical databases. The pathway analysis of identified potential biomarkers were performed by KEGG (http://www.kegg.com/, accessed on 5 May 2021), MetaboAnalyst 3.0 (http://www.metaboanalyst.ca, accessed on 20 June 2021) website and Cytoscape software.

## 5. Conclusions

In this study, a co-culture model of MCF-7 and HUVEC cells was investigated by metabolomics analysis based on UPLC-Q-TOF/MS. The results showed that HUVEC cells could induce the upregulation of glutathione metabolism, methionine cycle, purine metabolism, and TCA cycle in MCF-7 cells and promote the development of cancer cells to the malignant phenotype. The co-culture model was closer to the tumor real environment. In addition, it was found that AE and EMD could inhibit invasion and metastasis of MCF-7 cells, but their action stages were different. AE could inhibit the adhesion, invasion, and the secretion of VEGF and MMP-2, while EMD mainly inhibited adhesion and the secretion of MMP-2 and MMP-9. Furthermore, the mechanism of AE and EMD inhibiting metastasis was studied by metabolomics analysis. The results showed that AE significantly disturbed the metabolism of MCF-7 cells than EMD. AE had a good inhibitory effect on polyamine metabolism, methionine cycle, TCA cycle, glutathione metabolism, purine metabolism, and aspartate synthesis. EMD also showed inhibitory effects on these pathways, but its effect was weak. These metabolic pathways provide a new understanding of the mechanism of AE inhibition of breast cancer metastasis. At the same time, these metabolic pathways are also potential targets for clinical tumor therapy.

## Figures and Tables

**Table 1:** Identification and change trend of biomarkers.

Mode	Metabolites	RT (min)	Measured Mass (Da)	Mass Error (ppm)	AE	EMD	DOX	Pathway
ESI+	Spermine	0.50	203.2238	1.0	↓ *	-	↓ ***	Polyamine metabolism
	Arginine	0.65	174.1128	6.3	-	-	↓ **	Arginine biosynthesis
	Creatine	0.45	132.1000	−8.3	↓ *	-	↓ **	Glycine, serine, and threonine metabolism
	Methionine	1.12	150.0589	0.0	↓ *	-	↓ **	Methionine cycle
	Glutathione	1.39	308.0916	0.0	↓ **	↓ *	↓ **	Glutathione metabolism
	Isoleucine	1.93	132.1031	4.5	↑ ***	-	↑ ***	Valine, leucine, and isoleucine degradation
	2-phenylglycine	2.16	152.0700	4.8	-	-	↑ **	-
	Pyridoxine	2.16	170.0825	4.7	↑ *	-	↑ ***	Vitamin B6 metabolism
	Adenine	1.26	136.0630	5.1	↓ **	-	↓ ***	Purine metabolism
	ADP	4.47	428.0375	0.7	↓ *	-	↓ *	Purine metabolism
	Oxidized glutathione	4.47	613.1589	−1.5	↑ *	↑ *	↑ *	Glutathione metabolism
	Phenylalanine	4.70	166.0876	4.8	↑ ***	↑ *	↑ ***	Phenylalanine metabolism
	Guanine	4.70	152.0580	5.3	-	↑ **	-	Purine metabolism
	1H-indole-3-carboxaldehyde	5.29	146.0614	5.5	↑ *	-	↑ ***	Tryptophan metabolism
	Indoleacrylic acid	5.29	188.0723	5.8	↑ **	-	↑ ***	Tryptophan metabolism
	Phytosphingosine	9.54	318.3009	0.3	↑ ***	↑ *	↑ ***	Sphingolipid metabolism
ESI−	Glutamic acid	0.73	146.0455	1.4	-	-	↓ ***	D-glutamine and D-glutamate metabolism
	Tyrosine	2.49	180.0662	0.6	↑ ***	-	↑ ***	Tyrosine metabolism
	Xanthine	2.49	151.0257	0.7	↓ ***	-	↓ ***	Purine metabolism
	Glutathione	1.39	306.0759	−0.3	↓ *	↓ *	↓ ***	Glutathione metabolism
	Oxidized glutathione	4.47	611.1439	−0.3	↑ *	-	↓ *	Glutathione metabolism
	Malic acid	0.88	133.0130	−5.3	↓ ***	↓ *	↓ ***	Citrate cycle (TCA cycle)
	Phenylalanine	4.62	164.0720	4.8	↑ **	↑ *	↑ ***	Phenylalanine metabolism
	Hypoxanthine	1.93	135.0308	0.7	↓ ***	↓ *	↓ ***	Purine metabolism
	Tryptophan	5.29	203.0820	−0.5	↑ **	-	↑ ***	Tryptophan metabolism
	Pantothenic acid	4.96	218.1029	0.5	↑ ***	↓ *	↑ ***	Pantothenate and CoA biosynthesis
	Azelaic acid	7.12	187.0970	0.0	↑ *	-	↑ *	-
	S-adenosylhomo-cysteine	4.47	383.1134	−1.0	↓ *	↓ *	↓ ***	Methionine cycle
	Cytosine	0.88	110.0356	1.8	↓ *	-	↓ ***	Pyrimidine metabolism
	N-formyl-L-glutamic acid	1.26	174.0402	0.0	↓ ***	↑ ***	↓ ***	Histidine metabolism
	Folic acid	5.47	440.1315	−0.9	↑ **	-	↑ ***	Folate biosynthesis
	Inosine	4.62	267.0735	2.2	↑ *	-	↑ **	Purine metabolism
	Citric acid	1.72	191.0192	0.0	↑ *	-	↑ *	Citrate cycle (TCA cycle)
	Guanosine	4.62	282.0839	0.4	↓ *	↓ *	↓ *	Purine metabolism

* *p* < 0.05, ** *p* < 0.01, *** *p* < 0.001 compared with control group. ↑, ↓ represent the increase and decrease of metabolites compared with the control group, respectively.

## Data Availability

Not applicable.

## Supplementary Materials

The following supporting information can be downloaded at: https://www.mdpi.com/article/10.3390/ijms232213738/s1.

## References

[1] Fidler I.J. (2002). The organ microenvironment and cancer metastasis. Differentiation.

[2] Joyce J.A., Pollard J.W. (2009). Microenvironmental regulation of metastasis. Nat. Rev. Cancer.

[3] Stroka K.M., Konstantopoulos K. (2014). Physical Biology in Cancer. 4. Physical cues guide tumor cell adhesion and migration. Am. J. Physiol. Physiol..

[4] Cao Z., Ding B.S., Guo P., Lee S.B., Butler J.M., Casey S.C., Simons M., Tam W., Felsher D.W., Shido K. (2014). Angiocrine factors deployed by tumor vascular niche induce B cell lymphoma invasiveness and chemoresistance. Cancer Cell.

[5] Maishi N., Hida K. (2017). Tumor endothelial cells accelerate tumor metastasis. Cancer Sci..

[6] Chen Y.Y., Chiang S.Y., Lin J.G., Ma Y.S., Liao C.L., Weng S.W., Lai T.Y., Chung J.G. (2010). Emodin, aloe-emodin and rhein inhibit migration and invasion in human tongue cancer SCC-4 cells through the inhibition of gene expression of matrix metalloproteinase-9. Int. J. Oncol..

[7] Gu J., Cui C.F., Yang L., Wang L., Jiang X.H. (2019). Emodin inhibits colon cancer cell invasion and migration by suppressing epithelial-mesenchymal transition via the Wnt/beta-catenin pathway. Oncol. Res..

[8] Suboj P., Babykutty S., Gopi D.R.V., Nair R.S., Srinivas P., Gopala S. (2012). Aloe emodin inhibits colon cancer cell migration/angiogenesis by downregulating MMP-2/9, RhoB and VEGF via reduced DNA binding activity of NF-kappa B. Eur. J. Pharm. Sci..

[9] Sun Y., Wang X.F., Zhou Q.M., Lu Y.Y., Zhang H., Chen Q.L., Zhao M., Su S.B. (2015). Inhibitory effect of emodin on migration, invasion and metastasis of human breast cancer MDA-MB-231 cells in vitro and in vivo. Oncol. Rep..

[10] He Z.H., Huang Y.Q., Weng S.F., Tan Y.R., He T.P., Qin Y.M., Liang N.C. (2013). Effect of Aloe emodin on invasion and metastasis of high metastatic breast cancer MDA-MB-231 cells. J. Chin. Med. Mater..

[11] Lin M.L., Lu Y.C., Chung J.G., Wang S.G., Lin H.T., Kang S.E., Tang C.H., Ko J.L., Chen S.S. (2010). Down-Regulation of MMP-2 Through the p38 MAPK-NF-kappa B-Dependent Pathway by Aloe-Emodin Leads to Inhibition of Nasopharyngeal Carcinoma Cell Invasion. Mol. Carcinog..

[12] Manu K.A., Shanmugam M.K., Ong T.H., Subramaniam A., Siveen K.S., Perumal E., Samy R.P., Bist P., Lim L.H.K., Kumar A.P. (2013). Emodin Suppresses Migration and Invasion through the Modulation of CXCR4 Expression in an Orthotopic Model of Human Hepatocellular Carcinoma. PLoS ONE.

[13] Bansal A., Simon M.C. (2018). Glutathione metabolism in cancer progression and treatment resistance. J. Cell Biol..

[14] Sanderson S.M., Gao X., Dai Z., Locasale J.W. (2019). Methionine metabolism in health and cancer: A nexus of diet and precision medicine. Nat. Rev. Cancer.

[15] Morris J.P., Yashinskie J.J., Koche R., Chandwani R., Tian S., Chen C.C., Baslan T., Marinkovic Z.S., Sanchez-Rivera F.J., Leach S.D. (2019). alpha-Ketoglutarate links p53 to cell fate during tumour suppression. Nature.

[16] Platten M., Wick W., Van den Eynde B.J. (2012). Tryptophan catabolism in cancer: Beyond IDO and tryptophan depletion. Cancer Res..

[17] Zhou W., Yao Y., Scott A.J., Wilder-Romans K., Dresser J.J., Werner C.K., Sun H., Pratt D., Sajjakulnukit P., Zhao S.G. (2020). Purine metabolism regulates DNA repair and therapy resistance in glioblastoma. Nat. Commun..

[18] Chavarrıa T., Sanchez-Jimenez F., Quesada A.R., Medina M.A. (2003). Homocysteine inhibits the proliferation and invasive potential of HT-1080 human fibrosarcoma cells. Biochem. Biophys. Res. Commun..

[19] Ciccarone F., Vegliante R., Di Leo L., Ciriolo M.R. (2017). The TCA cycle as a bridge between oncometabolism and DNA transactions in cancer. Semin. Cancer Biol..

[20] Galluzzi L., Vacchelli E., Michels J., Garcia P., Kepp O., Senovilla L., Vitale I., Kroemer G. (2013). Effects of vitamin B6 metabolism on oncogenesis, tumor progression and therapeutic responses. Oncogene.

[21] Pedley A.M., Benkovic S.J. (2017). A New View into the Regulation of Purine Metabolism: The Purinosome. Trends Biochem. Sci..

[22] Tian Y., Du W., Cao S., Wu Y., Dong N., Wang Y., Xu Y. (2017). Systematic analyses of glutamine and glutamate metabolisms across different cancer types. Chin. J. Cancer.

[23] Casero R.A., Murray Stewart T., Pegg A.E. (2018). Polyamine metabolism and cancer: Treatments, challenges and opportunities. Nat. Rev. Cancer.

[24] Wang Z., Yip L.Y., Lee J.H.J., Wu Z., Chew H.Y., Chong P.K.W., Teo C.C., Ang H.Y., Peh K.L.E., Yuan J. (2019). Methionine is a metabolic dependency of tumor-initiating cells. Nat. Med..

[25] Sullivan L.B., Luengo A., Danai L.V., Bush L.N., Diehl F.F., Hosios A.M., Lau A.N., Elmiligy S., Malstrom S., Lewis C.A. (2018). Aspartate is an endogenous metabolic limitation for tumour growth. Nat. Cell Biol..

[26] Mierke C.T. (2011). Cancer Cells Regulate Biomechanical Properties of Human Microvascular Endothelial Cells. J. Biol. Chem..

[27] Kim A., Ma J.Y. (2019). Piceatannol-3-*O*-beta-d-glucopyranoside (PG) exhibits in vitro anti-metastatic and anti-angiogenic activities in HT1080 malignant fibrosarcoma cells. Phytomedicine.

[28] Deryugina E.I., Quigley J.P. (2015). Tumor angiogenesis: MMP-mediated induction of intravasation- and metastasis-sustaining neovasculature. Matrix Biol..

[29] Yao Z., Yuan T., Wang H., Yao S., Zhao Y., Liu Y., Jin S., Chu J., Xu Y., Zhou W. (2017). MMP-2 together with MMP-9 overexpression correlated with lymph node metastasis and poor prognosis in early gastric carcinoma. Tumor Biol..

[30] Zong L., Xing J., Liu S., Liu Z., Song F. (2018). Cell metabolomics reveals the neurotoxicity mechanism of cadmium in PC12 cells. Ecotoxicol. Environ. Saf..

