# Supplementary material for: Cell Metabolomics Reveals the Potential Mechanism of Aloe Emodin and Emodin Inhibiting Breast Cancer Metastasis
Source: Int J Mol Sci. 2022 Nov 8;23(22):13738. doi: 10.3390/ijms232213738 (PMC9694700; doi:10.3390/ijms232213738)
Supplement: Supplementary file 1 [file ijms-23-13738-s001.zip › ijms-1988233-supplementary.pdf]

Table S1. Identification of biomarkers between culture alone and co-culture MCF-7 cells.

Mode	Metabolites	RT (min)	Measured mass (Da)	Mass error (ppm)	Fragment ion (m/z)
ESI+	Arginine	0.65	174.1128	6.3	158, 130, 116, 112, 70, 60
	Methionine	1.12	150.0589	0.0	133, 104, 102, 87, 61, 56
	Glutathione	1.39	308.0916	0.0	290, 233, 179, 162, 144, 130, 116, 76
	2-Phenylglycine	2.16	152.0700	4.8	134, 106, 79
	Pyridoxine	2.16	170.0825	4.7	152, 134, 124, 106
	Adenine	1.26	136.0630	5.1	119, 109, 94
	ADP	4.47	428.0375	0.7	348, 136
	Oxidized glutathione	4.47	613.1589	−1.5	595, 538, 484, 409, 355
	Indoleacrylic acid	5.29	188.0723	5.8	170, 146, 118
	1H-Indole-3-carboxaldehyde	5.29	146.0614	5.5	118, 91
	Guanine	4.70	152.0580	5.3	135, 110
ESI-	Glutamic acid	0.73	146.0455	1.4	128, 102, 84
	Tyrosine	2.49	180.0662	0.6	163, 136, 119, 93
	Xanthine	2.49	151.0257	0.7	133, 108
	Glutathione	1.39	306.0759	−0.3	288, 272, 254, 210, 197, 179, 143, 128
	Oxidized glutathione	4.47	611.1439	−0.3	593, 567, 482, 306, 288, 272, 258, 242, 177, 160, 128
	Phenylalanine	4.62	164.0720	4.8	147, 103, 92, 72
	Hypoxanthine	1.93	135.0308	0.7	108, 92, 65
	Pantothenic acid	4.96	218.1029	0.5	146, 88, 71
	S-Adenosylhomocysteine	4.47	383.1134	−1.0	134, 107
	N-Formyl-L-glutamic acid	1.26	174.0402	0.0	146, 130, 102, 86
	Inosine	4.62	267.0735	2.2	135, 108, 92
	Citric acid	1.72	191.0192	0.0	173, 129, 111, 102, 87, 85
	Guanosine	4.62	282.0839	0.4	150, 133, 108
	Tryptophan	5.29	203.0820	−0.5	186, 159, 142, 130, 116, 74
	Malic acid	0.88	133.0130	−5.3	115, 89, 71
	Cytosine	0.88	110.0356	1.8	67, 42

Table S2. Multiple reaction monitoring (MRM) parameters for metabolites.

Analytes	Adduct Ion	MRM transition (m/z)	Q1 Pre deviation (V)	CE (V)	Q3 Pre deviation (V)
Methionine	$[M + H]^+$	150.00 > 56.05	−20	−17	−20
		150.00 > 104.10	−26	−13	−20
SAM	$[M-H]^-$	398.95 > 96.80	12	35	13
		398.95 > 171.20	28	31	16
SAH	$[M + H]^+$	385.10 > 136.05	−14	−20	−24
		385.10 > 134.10	−14	−20	−28
Homocysteine	$[M + H]^+$	136.00 > 90.15	−29	−12	−17
		136.00 > 56.15	−22	−18	−22
Cysteine	$[M + H]^+$	122.05 > 59.10	−30	−23	−22
		122.05 > 76.10	−30	−15	−29
Glutathione	$[M-H]^-$	306.10 > 272.20	15	13	28
		306.10 > 143.20	15	19	14
Oxidized glutathione	$[M-H]^-$	611.10 > 306.20	28	25	21
		611.10 > 272.25	28	29	17
Glutamate	$[M + H]^+$	148.05 > 84.15	−17	−16	−20
		148.05 > 56.15	−10	−27	−22
Glutamine	$[M + H]^+$	147.05 > 84.05	−20	−20	−20
		147.05 > 130.00	−20	−15	−20
Fumarate	$[M-H]^-$	115.25 > 71.00	13	12	12
		115.25 > 27.10	13	11	10
Malate	$[M-H]^-$	133.20 > 115.05	27	16	11
		133.20 > 71.00	27	16	10
Succinate	$[M-H]^-$	117.20 > 73.10	25	14	11
		117.20 > 99.90	13	24	15
Citrate	$[M-H]^-$	191.10 > 111.05	15	13	10
		191.10 > 87.05	21	21	13
α-Ketoglutarate	$[M-H]^-$	145.10 > 101.10	15	12	10
		145.10 > 57.05	15	13	22
Aspartate	$[M + H]^+$	134.10 > 74.10	−22	−15	−29
		134.10 > 88.15	−22	−12	−16
Inosine	$[M + H]^+$	269.05 > 137.05	−20	−10	−20
		269.05 > 119.15	−10	−38	−22
Hypoxanthine	$[M + H]^+$	137.10 > 119.15	−20	−22	−12
		137.10 > 55.05	−21	−30	−21
Xanthosine	$[M + H]^+$	285.05 > 153.05	−20	−11	−20
		285.05 > 136.00	−13	−29	−14
Xanthine	$[M + H]^+$	153.00 > 71.20	−11	−19	−29
		153.00 > 91.65	−16	−13	−22
Guanosine	$[M + H]^+$	284.05 > 152.10	−20	−20	−20
		284.05 > 135.15	−10	−35	−14

Tryptophan	$[M + H]^+$	205.15 > 188.05	−20	−10	−20
		205.15 > 118.00	−20	−25	−20
Pyridoxal-5′-phosphate	$[M + H]^+$	247.95 > 150.10	−20	−16	−20
		247.95 > 94.20	−18	−28	−19
N, N-Phe	$[M + H]^+$	194.25 > 148.10	−20	−15	−20
		194.25 > 133.05	−20	−25	−20

Table S3. Regression equation, correlation coefficient and linear range of metabolites.

Analytes	Linear equation	R^2	Dynamic range (ng/mL)
Methionine	y = 1.0517x-0.0497	0.9918	0.2–4000
SAH	y = 0.0135x + 0.0007	0.9992	4–8000
Homocysteine	y = 0.5600x − 0.0232	0.9941	2–4000
Cysteine	y = 0.0158x − 0.0036	0.9915	40–4000
Glutathione	y = 199.044x + 7396.65	0.9990	20–10,000
Oxidized glutathione	y = 619.718x-8882.17	0.9996	8–8000
Glutamate	y = 0.0187x + 0.0055	0.9921	4–8000
Fumarate	y = 22.7573x + 12,372.3	0.9971	40–10,000
Malate	y = 810.787x + 20,233.0	0.9997	4–10,000
Succinate	y = 88.6555x + 4942.38	0.9950	40–8000
Citrate	y = 465.964x + 16147.2	0.9980	0.4–40,000
α-Ketoglutarate	y = 214.708x + 4383.15	0.9908	200–10,000
Inosine	y = 0.0802x + 0.0009	0.9998	0.1–4000
Hypoxanthine	y = 0.0298x − 0.0034	0.9972	10–10,000
Xanthosine	y = 0.1839x + 0.0061	0.9991	0.1–10,000
Xanthine	y = 183.257x − 19,362.2	0.9946	10–10,000
Guanosine	y = 0.0984x − 0.0017	0.9992	0.1–10,000
Tryptophan	y = 0.5269x-0.0076	0.9997	0.01–8000

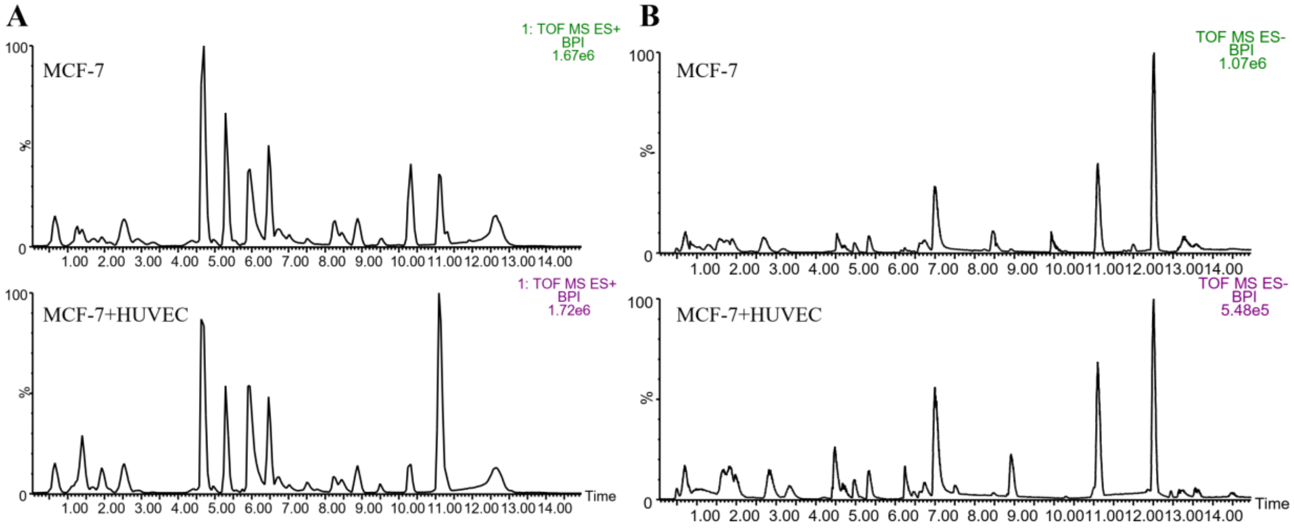

Figure S1. Extracted ion chromatograms of polar metabolites of culture alone and co-culture MCF-7 cells in positive (**A**) and negative(**B**) ion modes.

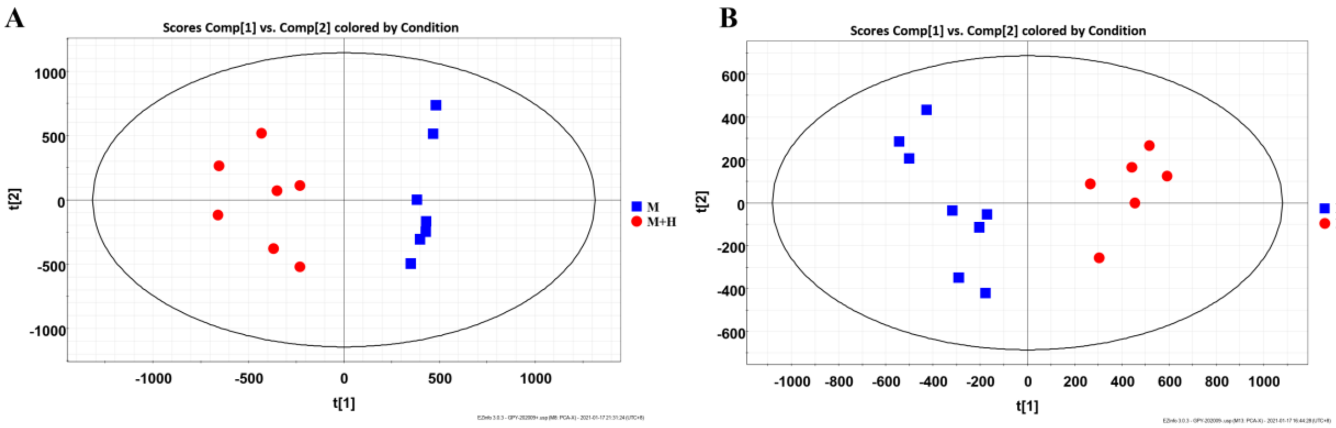

Figure S2. PCA score plots of polar metabolites of MCF-7 cells in positive (A) and negative (B) ion modes.

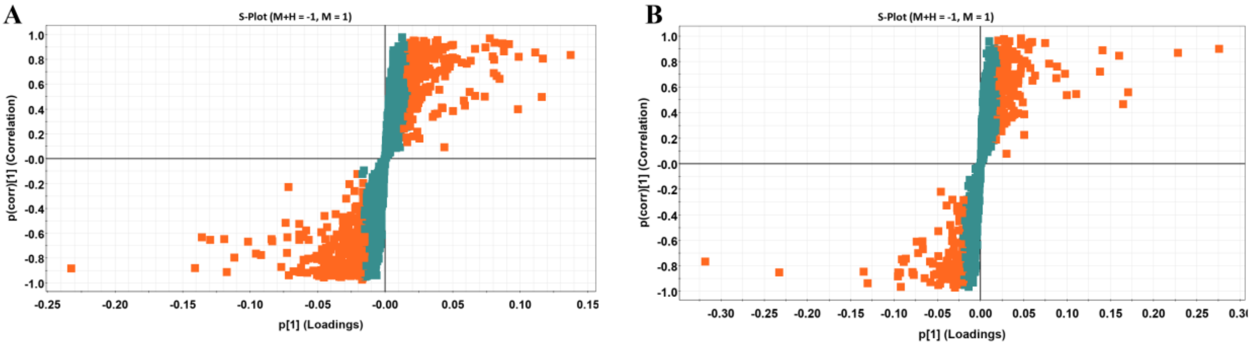

Figure S3. S-plots obtained by OPLS-DA analysis of polar metabolites between culture alone and co-culture MCF-7 cells in positive (**A**) and negative (**B**) ion modes.

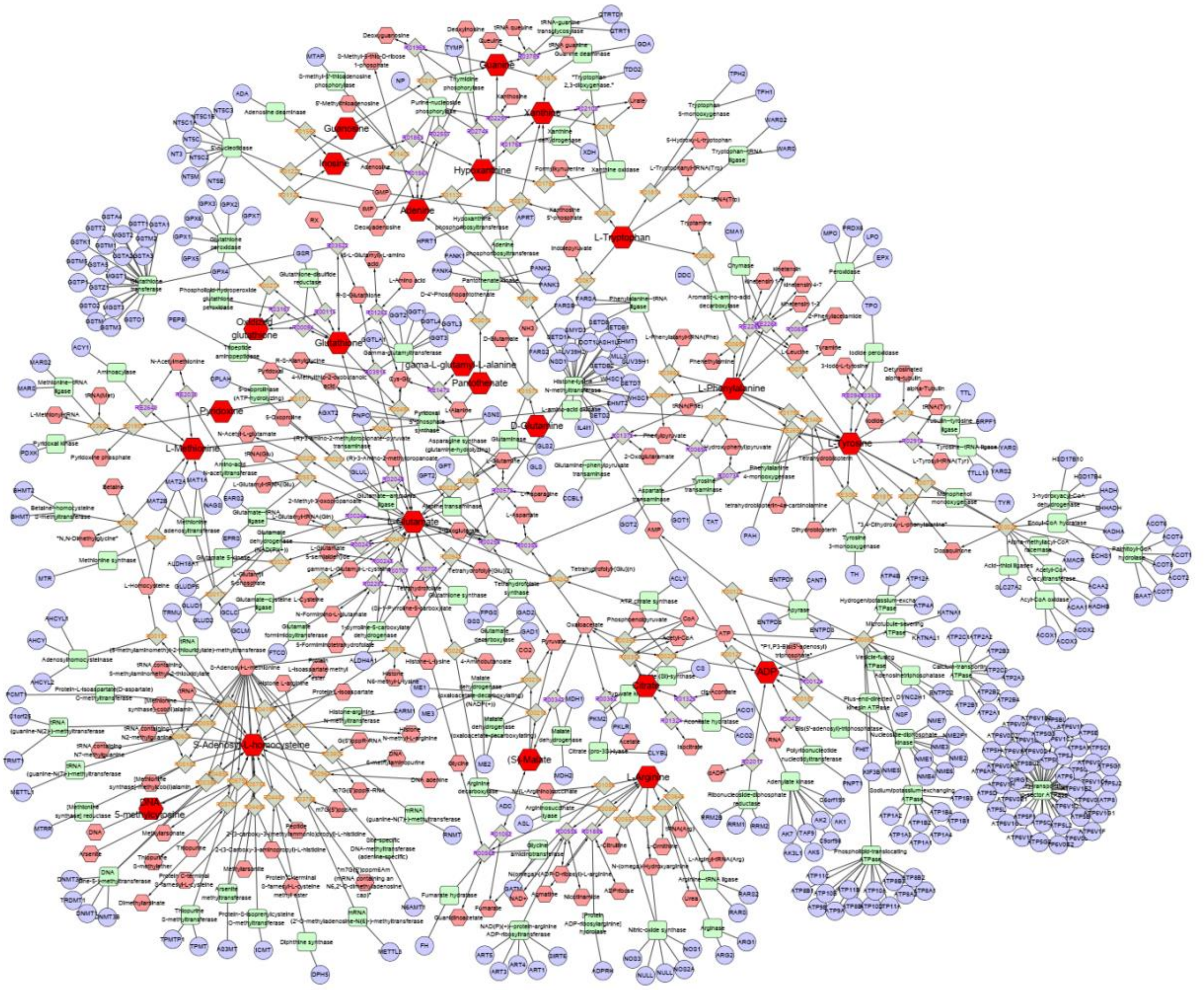

Figure S4. Gene-enzyme-biomarker network built by Cytoscape of potential biomarkers which were filtered out in this study.

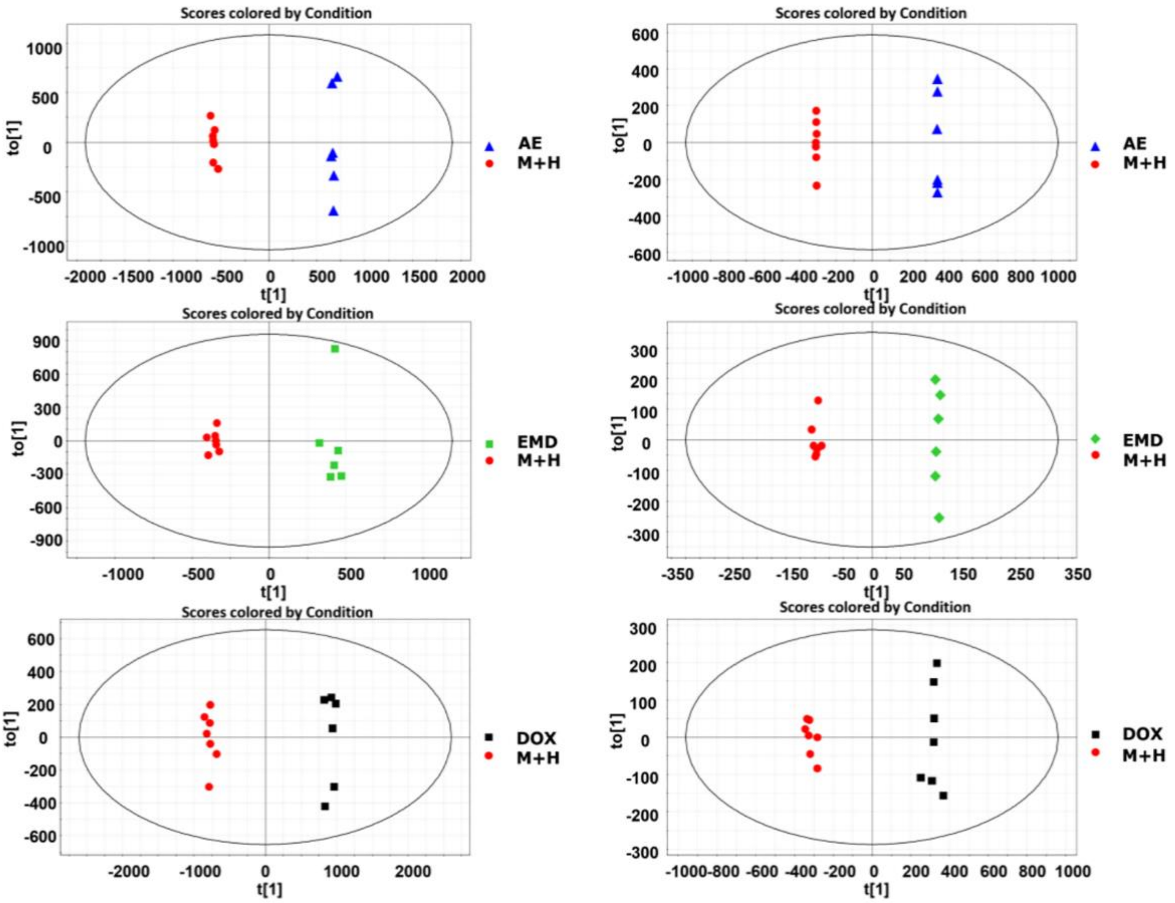

Figure S5. PLS-DA score plots of metabolic profiling between aloe emodin (AE)/emodin (EMD)/doxorubicin (DOX) and control group (M + H) in positive (left) and negative (right) ion modes.
